# Physical inactivity in older adults with cognitive impairment without dementia: room for improvement

**DOI:** 10.1007/s40520-021-01999-5

**Published:** 2021-10-21

**Authors:** Matthew J. Miller, Irena Cenzer, Deborah E. Barnes, Kenneth E. Covinsky

**Affiliations:** a Department of Physical Therapy and Rehabilitation Science, University of California, San Francisco, San Francisco, CA, USA; b Division of Geriatrics, University of California, San Francisco, San Francisco, CA, USA; c San Francisco VA Health Care System, San Francisco, CA, USA; d Department of Psychiatry and Behavioral Sciences, University of California, San Francisco, San Francisco, CA, USA; e Department of Epidemiology & Biostatistics, University of California, San Francisco, San Francisco, CA, USA

**Keywords:** cognitive impairment, physical activity, physical function, disability

## Abstract

**Background:**

Persons with cognitive impairment without dementia are at high risk of adverse health outcomes. Tailored intervention targeting moderate-vigorous physical activity (MVPA) may reduce these risks.

**Aims:**

To identify the prevalence and predictors of physical inactivity among older adults with cognitive impairment, no dementia (CIND); and estimate the proportion of inactive people with CIND who are capable of greater MVPA.

**Methods:**

We studied 1875 community dwelling participants (over age 65) with CIND in the Health and Retirement Study. Physical inactivity was defined as MVPA ≤1x/week. Associations of physical inactivity with sociodemographic, health, and physical function were examined using chi-square and modified Poisson regression. We considered physically inactive participants capable of greater MVPA if they reported MVPA at least 1-3x/month, no difficulty walking several blocks, or no difficulty climbing several flights of stairs.

**Results:**

Fifty-six percent of participants with CIND were physically inactive. Variables with the highest age, sex, and race/ethnicity adjusted risk ratio (ARR) for physical inactivity were self-rated health (poor [76.9%]vs. excellent[34.2%]; ARR[95% CI]: 2.27[1.56–3.30]), difficulty walking (across the room [86.5%]vs. none[40.5%]; ARR[95% CI]:2.09[1.87–2.35]), total assets (lowest quartile [62.6%]vs. highest quartile[43.1%]; ARR[95% CI]:1.54[1.29–1.83]), and lower education attainment (less than high school [59.6%]vs college graduate[42.8%]; ARR[95% CI]:1.46[1.17–1.83]). Among physically inactive older adults with CIND, 61% were estimated to be capable of greater MVPA.

**Conclusions:**

Although physical inactivity is prevalent among older adults with CIND, many are capable of greater MVPA. Developing tailored physical activity interventions for this vulnerable population may improve cognitive, health, and quality of life outcomes.

## Introduction

There are nearly 6 million Americans living with Alzheimer’s disease, and the prevalence is expected to rise to 14 million by 2050.[1] More than 10 million older adults in the United States have cognitive impairment that is not severe enough to qualify for a diagnosis of dementia,[2] a condition generally referred to as mild cognitive impairment (MCI) in clinical settings or cognitive impairment, no dementia (CIND) in epidemiologic studies.[3,4] While an estimated 38% of these persons progress to dementia within 5 years, many have cognitive function that will remain stable or even improve.[5,6] Beyond risk of further cognitive decline, older adults with CIND are at greater risk of incident disability and mortality compared to those without CIND.[7] The absence of pharmacologic therapies that effectively reduce the risk of CIND progressing to dementia highlights the potential importance of non-pharmacologic interventions in improving health outcomes and quality of life in this vulnerable population.[8–12]

Approximately 40% of Alzheimer’s disease cases may be attributable to modifiable factors like physical inactivity.[8,10,12] Physical inactivity is estimated to account for more than 20% of Alzheimer’s disease cases in the United States,[8,13] and greater physical activity among older adults has beneficial effects on all-cause mortality, fall risk, physical function, maintenance of independence, and mental health.[14,15] Despite the potential benefits of regular physical activity, less than 50% of community dwelling older adults >65 years old adhere to moderate-vigorous physical activity (MVPA) guidelines.[16] Poor MVPA adherence and resulting physical inactivity among older adults is multifactorial and encompasses a wide range of individual characteristics including age, race/ethnicity, body mass index, self-rated health, mental health, and physical function.[17] Interventions tailored to individual characteristics, needs, and goals may result in greater physical activity among community dwelling older adults.[18]

Older adults with CIND likely represent a high risk population where tailored physical activity intervention is needed to improve cognitive and health outcomes.[19] Physical activity interventions have been developed for community dwelling older adults because individual characteristics that are associated with physical inactivity have been identified for this population. Characteristics associated with physical inactivity have not specifically been investigated in older adults with CIND, therefore few interventions tailored to this specific population have been developed. Further, the size of the population of older adults with CIND who are capable of greater MVPA, and arguably most prepared for physical activity intervention, is unknown. This formative knowledge is needed to support the development of physical activity interventions that are tailored to the unique needs of older adults with CIND. Therefore, the purpose of this study was to identify the prevalence and predictors of physical inactivity among older adults with CIND. An additional purpose of this study was to estimate the proportion of people with CIND who are capable of greater MVPA.

## Methods

### Data sources

We used data from the 2016 wave of the Health and Retirement Study (HRS). Details of the HRS have been documented elsewhere.[20] Briefly, the HRS is a longitudinal, nationally representative cohort study of adults over 50 years old conducted by the University of Michigan and supported by the National Institute of Aging and Social Security Administration. Participants are selected using a multistage probability sampling design and trained interviewers collected data from participants in a variety of topic areas (e.g., cognition, finances, health, functional limitation) using face-to-face or telephone methods.

### Participants

Community dwelling participants in the 2016 wave of HRS were included in this study if they were 65 years or older and had CIND. Participants were excluded if they were living in a nursing home or if responses were provided by proxy. Inclusion and exclusion criteria were selected achieve the study purpose and generate knowledge to support the development of interventions tailored to the unique needs of older adults with CIND.

We identified CIND using a validated method that has been used in multiple HRS studies.[21,22] We used the HRS cognitive scale (score range: 0 - 27), which consists of tests of immediate and delayed recall of ten common nouns, serial subtractions by seven, and a backward count task from 20. [21,22] Cognitive scale cutpoints for normal cognition, CIND, and Dementia were validated using data from the Aging, Demographics, and Memory Study (ADAMS). The ADAMS was designed to investigate the epidemiology of Alzheimer’s disease and dementia within HRS.[22,23] Participants with a score within the range of 7 to 11 points indicated the presence of CIND, and were included in this study. Of the 19046 community-dwelling, self-respondent participants in the 2016 wave of HRS, 9959 were excluded because they were under the age of 65. Of the remaining 9087 participants, 6641 (73%) scored 12 or more on the cognitive scale and were classified as having normal cognition and 553 (6%) scored 6 or less and were classified as having dementia. The 1893 (21%) participants who scored 7-11 were classified as having CIND were potential participants for this study. Of these, we excluded 18 participants for whom severity of walking or stair climbing difficulty could not be determined, leaving a final cohort of 1875 participants.

### Measures

#### Physical inactivity:

We used two questions to determine self-reported MVPA. Moderate physical activity was assessed by asking participants, “How often do you take part in sports or activities that are moderately energetic, such as gardening, cleaning the car, walking at a moderate pace, dancing, floor or stretching exercises?” Vigorous physical activity was assessed by asking participants: “How often do you take part in sports or activities that are vigorous, such as running or jogging, swimming, cycling, aerobics or gym workout, tennis, or digging with a spade or shovel?” For both questions, participants responded with more than once per week, once per week, one to three times per month, or never. Evidence suggests older adults who are physically inactive and/or sedentary are at greatest risk for progression of cognitive decline;[24,25] therefore, we defined physical inactivity as reporting a total of ≤ 1x/week of MVPA.

#### Predictor Variables:

Sociodemographic variables included age, gender, race/ethnicity, living alone, marital status, education, and total assets (participants divided into quartiles).

Health variables included body mass index, presence of health conditions, self-rated health, depression, current smoking, and current alcohol drinking. The presence of health conditions was assessed by asking participants if a doctor had ever told them they have high blood pressure, diabetes, cancer, lung disease, heart disease, stroke, or arthritis. We created a count of the number of conditions participants reported. Participant self-rated health was reported as excellent, very good, good, fair, or poor. The presence of depression was indicated by a score of >2 using the short Center for Epidemiological Studies Depression (CES-D) scale.[26]

Physical function variables included the presence of significant pain, severity of walking difficulty, and severity of stair climbing difficulty. The presence of significant pain was determined by asking participants that indicated they were often troubled with pain, “How bad is the pain most of the time: mild, moderate or severe?” Participants who responded “moderate” or “severe” were classified as experiencing significant pain.[27,28] We identified severity of walking difficulty based on participant responses to three yes/no questions. Participants were asked, “Because of a health problem do you have any difficulty with walking [several blocks, one block, across the room]?” We identified severity of stair climbing difficulty based on participant responses to two yes/no questions: “Because of a health problem, do you have difficulty with [climbing several flights of stairs without resting, climbing one flight of stairs without resting]?”.

#### Capability of greater MVPA:

Among participants who were physically inactive, we identified those who were capable of greater MVPA. Infrequent MVPA is an indicator of capability for regular MVPA, but at a frequency that is too low to achieve substantial health benefits. Additionally, those with adequate levels of physical function show the capacity required to engage in MVPA. Therefore, we categorized participants as capable of greater MVPA if they met any of the following criteria: 1) MVPA at least 1-3 times a month; 2) no difficulty walking several blocks; or 3) no difficulty climbing several flights of stairs.

### Analysis

We estimated the prevalence of sociodemographic, health, and physical function variables for the full analytic sample. Next, we assessed the associations of physical inactivity with sociodemographic, health, and physical function variables using chi-square tests. We then used modified Poisson regression models to calculate age, sex, and race/ethnicity adjusted risk ratios (ARR) and 95% confidence intervals (CI) for predictor variables.

Finally, we estimated the prevalence of capability for greater MVPA among older adults with CIND who were physically inactive. Predictors of physical inactivity may also influence capability; therefore, we also estimated prevalence of physically inactive participants who were capable of greater MVPA by stratifying by BMI (normal, underweight, overweight, obese), number of self-reported health conditions (0, 1 or 2, >2), depression status (no depression, depression), self-rated health (excellent, very good, good, fair, poor), and significant pain (no significant pain, significant pain). Statistical analyses accounted for the complex sampling design and differential probability of selection in HRS and were conducted using Stata software (StataCorp, College Station, version 16.1)

## Results

Participants had a mean (SD) age of 77 (7.6) years, were 58.2% female, 56.7% non-Hispanic White, 25.0% Black, and 55.4% high school graduates or equivalent (Table 1). The median (Quartile 1[Q1] – Quartile 3 [Q3]) cognitive score was 10 (Q1-Q3: 8-11) and count of health conditions was 3 (Q1-Q3: 2 – 4). Prevalence of walking difficulty for several blocks was 20.4%, one block was 15.5%, and across the room was 12.6%. Prevalence of difficulty climbing several flights of stairs was 30.6% and difficulty climbing one flight was 32.7%.

Fifty six percent of people with CIND reported MVPA ≤1x/week and were classified as being physically inactive. Associations of physical inactivity with sociodemographic, health, and function variables are presented in Table 2. Variables with the strongest associations with being physically inactive included self-rated health (poor [76.9%] vs. excellent [34.2%]; ARR [95% CI]: 2.27 [1.56 – 3.30]), difficulty walking (across the room [86.5%] vs none [40.5%]; ARR [95% CI]: 2.09 [1.87 – 2.35]), total assets (lowest quartile [62.6%] vs highest quartile [43.1%]; ARR [95% CI]:1.54 [1.29–1.83]), and lower education attainment (less than high school [59.6%] vs college graduate [42.8%]; ARR [95% CI]: 1.46 [1.17 – 1.83]).

**Figure 1.**
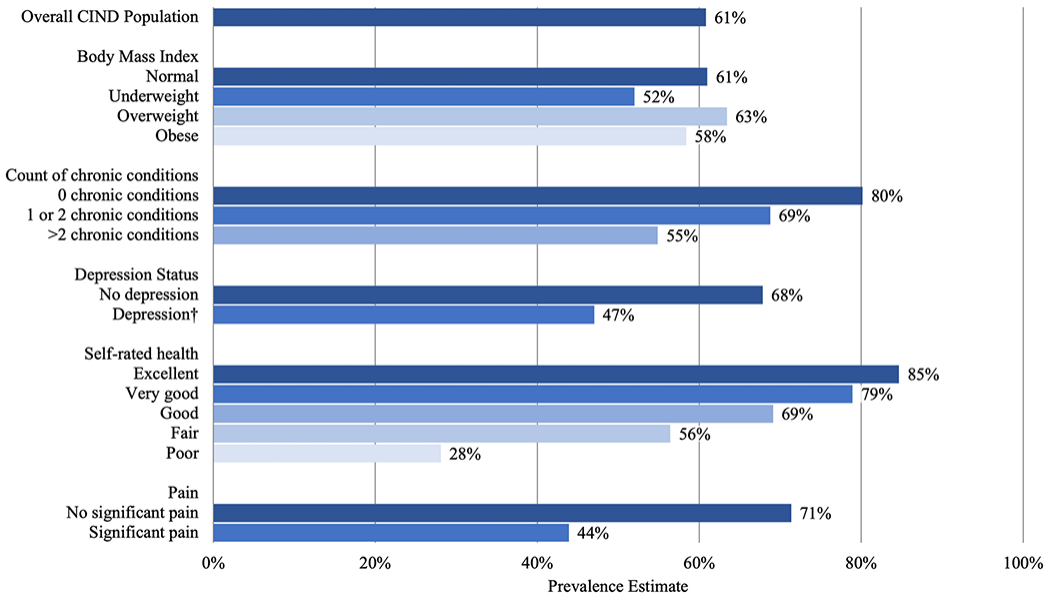
Estimated population prevalence of physically inactive people with CIND (moderate-vigorous physical activity [MVPA] ≤1x/week) who are capable of greater MVPA. We defined capable as a report of moderate or vigorous physical activity at least 1-3x/month, or no difficulty with stair climbing, or no difficulty with walking multiple blocks. CIND: Cognitive impairment, no dementia; BMI: Normal: 18.5 – 24.9 kg/m^2^, Underweight: < 18.5 kg/m^2^, Overweight: 25 – 9.9 kg/m^2^, Obese: >30 kg/m^2^; † Center for Epidemiological Studies Depression Scale score >2 point indicated depression

Among older adults with CIND who were physically inactive, 61% were estimated to be capable of greater MVPA (Figure 1). Considering the three criteria to determine capability of greater MVPA, an estimated 41.8% reported MVPA at least 1-3x/month, 38.1% reported no difficulty with walking several blocks, and 26.9% reported no difficulty with climbing several flights of stairs. More than two-thirds of older adults were estimated to be capable if they reported no depression (68%), no significant pain (71%), no chronic health conditions (80%), 1 or 2 chronic health conditions (69%), and self-rated health of good (69%), very good (79%), or excellent (85%). A substantial number of physically inactive older adults with CIND were capable of greater MVPA even when reporting poor self-rated health (28%), significant pain (44%), or depression (47%).

## Discussion

Physical activity is a modifiable factor that may confer clinically meaningful benefits for the vulnerable population of older adults with CIND. [8,10,12] The purpose of the present study was to identify the prevalence and predictors of physical inactivity among older adults with CIND. We found that 56% percent of older adults with CIND were physically inactive and that 61% of these inactive older adults are likely capable of greater MVPA. Importantly, a large proportion of older adults with CIND who had characteristics that were associated with physical inactivity were also capable of greater MVPA. Thus, developing tailored physical activity interventions for older adults with CIND may have a significant impact on cognitive and health outcomes.

Prior research suggests that a range of sociodemographic, health, and physical function characteristics are associated with MVPA adherence in the general population of community dwelling older adults.[17] In the present study, we identified predictors of physical inactivity in the specific population of older adults with CIND. Participants who reported poor self-rated health or difficulty walking across the room were approximately twice as likely to be physically inactive compared to those with excellent self-rated health or no difficulty walking. Remaining variables with significant, yet modest, associations with physical inactivity are put into context when considering the high prevalence of physical inactivity across reference groups. For example, 43% of participants who graduated from college were physically inactive compared to 60% among those with less than high school education, resulting in an ARR (95% CI) of 1.46 (1.17 – 1.83).

In addition to identifying predictors of physical inactivity, our findings suggest many physically inactive older adults with CIND are capable of greater MVPA. Although participants who reported no chronic health conditions, excellent self-rated health, or no significant pain were more likely to report MVPA more than one time per week, 71% - 85% of physically inactive participants who reported these same characteristics were identified as being capable of greater MVPA. While those with poorer health were less likely to demonstrate capability for greater MVPA, there continued to be a substantial proportion of older adults with CIND who were capable of greater MVPA despite reporting >2 chronic conditions, depression, poor self-rated health, and significant pain.

Engaging in greater MVPA is associated with improvements in health outcomes for older adults, especially those with cognitive impairment.[15,24,29–31] Exercise, among other risk-reduction targets (e.g., vascular control, nutrition, cognitive training), is also a core component of many large-scale multidomain dementia prevention clinical trials. [32–34] Community dwelling older adults who adhere to aerobic exercise guidelines, engaging in MVPA at least three days a week, are at a 32% lower risk of incident dementia compared to older adults who do not.[35] Further, older adults who start engaging in at least one day/week of MVPA following diagnosis of mild cognitive impairment have an estimated 11% reduction in risk for Alzheimer’s disease. [25] In addition to potential cognitive benefits, exercise has beneficial effects on physical function and quality of life among older adults with CIND.[36] The high prevalence of capability and potential benefits of any increases in MVPA suggests that physical activity promotion efforts may have significant clinical implications within this target population.

Many predictors of physical inactivity are common across the general population of older adults, yet current approaches may not address the unique contextual factors that influence engagement in exercise for older adults with CIND. For example, mobility limitations are a common barrier to physical activity among older adults;[17] yet, the reliance on a caretaker and stigma surrounding memory loss are unique to older adults with MCI or early dementia. [19] Motivation to participate in an exercise program is reduced if exercise programs do not accommodate for these unique contextual factors (e.g., memory loss, care taker).[19] Therefore, developing exercise programs and interventions that are inclusive of older adults with CIND, accommodate for the reliance on a caretaker, and use social engagement may increase motivation for adherence to MVPA in older adults with CIND. Finally, the high prevalence of self-reported physical function in this unique population is consistent with prior research,[37,38] and may highlight the role of rehabilitation professionals (e.g., physical therapists) to increase physical activity. Rehabilitation professionals can develop individualized exercise programs grounded in behavior change frameworks, target perceived barriers, and engage social support in an effort to increase MVPA and quality of life.[39]

There are limitations in the present study that should be acknowledged. This study was focused on community-dwelling older adults with CIND and may have limited generalizability to other populations (e.g., older adults with dementia). The use of self-reported physical activity may introduce recall bias in this study sample. Additionally, we were unable to estimate provide more detailed estimates of MVPA frequency due to limitations in HRS response options. Finally, regular physical activity at lower intensities may also confer benefits in older adults, but were not specifically assessed within this study.

## Conclusions

Fifty-six percent of community dwelling older adults with CIND are physically inactive, reporting MVPA ≤1x/week. Worse health and physical function, lower socioeconomic status, and lower educational attainment were risk factors of physical inactivity. An estimated 61% of those who were physically inactive were capable of greater MVPA. The high prevalence of physical inactivity, despite capability for greater MVPA, suggests developing and testing tailored physical activity interventions for older adults with CIND may be an important avenue for improving cognitive, health, and quality of life outcomes in this vulnerable group.

## Figures and Tables

**Table 1.** Characteristics of community dwelling older adults with CIND (N = 1875).

Characteristics		N	%
Age	<70	395	21.1
	70-80	788	42.0
	>80	692	36.9

Gender	Male	784	41.8
	Female	1091	58.2

Ethnicity	Non-Hispanic White	1063	56.7
	Black	468	25.0
	Hispanic	293	15.6
	Other	51	2.7

Live alone		614	32.8

Married/Partnered	Yes	877	46.8
	No	996	53.2

Total Assets	> $304,000	468	25.0
	$95,000 - $304,000	469	25.0
	$10 000 - $95,000	462	24.6
	<$10,000	476	25.4

Education	College graduate	177	9.4
	High school graduate or equivalent	1039	55.4
	Less than high school	659	35.2

Body Mass Index*	Normal	592	31.6
	Underweight	44	2.4
	Overweight	713	38.0
	Obese	570	30.4

Self-reported health conditions	High blood pressure	1422	75.9
	Diabetes	665	35.6
	Cancer	394	21.1
	Lung disease	287	15.4
	Heart disease	694	37.2
	Stroke	273	14.6
	Arthritis	1380	73.6

Count of health conditions	0	83	4.4
	1 or 2	744	39.7
	>2	1048	55.9

Depression^†^		534	28.5

Self-Reported Health	Excellent	91	4.9
	Very good	365	19.5
	Good	592	31.6
	Fair	611	32.6
	Poor	214	11.4

Current cigarette smoker		177	9.5

Current alcohol drinker		753	40.2

Significant pain		584	31.4

Walking difficulty	None	966	51.5
	Several blocks	382	20.4
	One block	291	15.5
	Across room	236	12.6

Stair climb difficulty	None	689	36.8
	Several flights	573	30.6
	One flight	613	32.7

CIND: Cognitive impairment no dementia;

* Normal: 18.5 – 24.9 kg/m^2^. Underweight: < 18.5 kg/m^2^. Overweight: 25 – 9.9 kg/m^2^. Obese: >30 kg/m^2^

† Center for Epidemiological Studies Depression Scale score >2 point indicated depression

**Table 2.** Associations of sociodemographic, health, and function variables with physical inactivity^¥^ (N = 1875)

Variables		Physical inactivity N (%)	P-Value	Adjusted Risk Ratio* (95% CI)
Age	<70 years	204 (50.4)	0.02	Reference
	70-80 years	444 (56.0)		1.14 (0.98 – 1.32)
	>80 years	420 (61.0)		1.22 (1.06 – 1.42)

Gender	Men	408 (51.7)	0.003	Reference
	Women	660 (60.0)		1.16 (1.06 - 1.28)

Race/ethnicity	Non-Hispanic White	621 (56.8)	0.24	Reference
	Black	269 (59.5)		1.08 (0.94 - 1.25)
	Hispanic	152 (50.4)		0.89 (0.77 - 1.03)
	Other	26 (48.9)		0.88 (0.65 - 1.19)

Lives alone	No	715 (56.3)	0.05	Reference
	Yes	353 (56.4)		0.95 (0.85 - 1.07)

Married/Partnered	Yes	469 (53.6)	0.10	Reference
	No	598 (58.9)		1.03 (0.91 - 1.16)

Total Assets	> $304,000	206 (43.1)	<.001	Reference
	$95,000 - $304,000	270 (58.1)		1.39 (1.18 - 1.63)
	$10 000 - $95,000	297 (64.7)		1.60 (1.35 - 1.91)
	<$10,000	295 (62.6)		1.54 (1.29 - 1.83)

Education	College graduate	80 (42.8)	0.01	Reference
	High school graduate / equivalent	606 (57.1)		1.36 (1.07 - 1.71)
	Less than high school	382 (59.6)		1.46 (1.17 - 1.83)

Body Mass Index	Normal	315 (51.5)	0.006	Reference
	Underweight	32 (65.3)		1.28 (0.94 - 1.76)
	Overweight	394 (55.0)		1.14 (1.01 - 1.28)
	Obese	359 (63.4)		1.33 (1.17 - 1.50)

Count of health conditions	0	36 (47.9)	<.001	Reference
	1 or 2	377 (48.4)		1.00 (0.74 - 1.35)
	>2	655 (63.0)		1.28 (0.97 - 1.70)

Depression^†^	No	702 (52.0)	<.001	Reference
	Yes	365 (67.5)		1.31 (1.20 - 1.43)

Self-rated health	Excellent	32 (34.2)	<.001	Reference
	Very good	161 (41.5)		1.21 (0.80 - 1.82)
	Good	324 (56.3)		1.65 (1.14 - 2.40)
	Fair	377 (63.1)		1.88 (1.28 - 2.75)
	Poor	172 (76.9)		2.27 (1.56 - 3.30)

Cigarette smoker	No	945 (55.4)	0.08	Reference
	Yes	116 (65.6)		1.26 (1.05 - 1.51)

Alcohol drinker	No	686 (61.3)	0.001	Reference
	Yes	380 (49.6)		0.82 (0.73 - 0.93)

Significant pain	No	666 (50.9)	<.001	Reference
	Yes	393 (68.4)		1.33 (1.23 - 1.44)

Walking difficulty	None	398 (40.5)	<.001	Reference
	Several blocks	243 (64.0)		1.55 (1.38 - 1.74)
	One block	230 (79.0)		1.91 (1.67 - 2.18)
	Across room	197 (86.5)		2.09 (1.87 - 2.35)

Stair climb difficulty	None	283 (40.3)	<.001	Reference
	Several flights	328 (56.5)		1.39 (1.21 - 1.60)
	One Flight	457 (75.1)		1.83 (1.63 - 2.06)

¥ Physical inactivity: Moderate Vigorous Physical Activity ≤1x/week

* modified Poisson regression model adjusted for age, sex, race/ethnicity

Normal: 18.5 – 24.9 kg/m^2^. Underweight <18.5 kg/m^2^. Overweight: 25 – 9.9 kg/m^2^, Obese: >30 kg/m^2^

† Center for Epidemiological Studies Depression Scale score >2 point indicated depression
